# Modified median hepatic fissure approach for resection of liver tumours located in the angle between the root of the middle and right hepatic veins

**DOI:** 10.1186/s12893-021-01412-y

**Published:** 2021-12-03

**Authors:** Hongcheng Lu, Linquan Wu, Rongfa Yuan, Wenjun Liao, Jun Lei, Jianghua Shao

**Affiliations:** Department of Hepatobiliary Surgery, Second Affiliated Hospital of Nanchang University, No. 1, Minde Road, Jiang Xi 330006 Nanchang, China

**Keywords:** Liver segment VIII tumour, Median hepatic fissure approach, Surgical treatment

## Abstract

**Background:**

Liver tumours between the root angle of the middle and right hepatic veins are a special type of liver segment VIII tumour. In this study, we designed a modified median hepatic fissure approach to remove these tumours. The safety and effectiveness of the approach were evaluated.

**Materials and methods:**

From April 2015 to November 2019, 11 patients with liver tumours between the angle of the middle and right hepatic veins underwent this modified median hepatic fissure approach. We retrospectively analysed data from the perioperative periods of these 11 patients, including general condition, operation time, intraoperative bleeding, and postoperative complications. Disease-free survival and overall survival were assessed.

**Results:**

Of the 11 patients, 9 patients had primary hepatocellular carcinoma and 2 had colorectal liver metastases. The average intraoperative blood loss was 285 mL (150–450 mL). Two patients developed postoperative bile leakage, but there were no significant serious complications, such as intraabdominal bleeding and liver failure, in any of the patients. The liver function returned to the normal range on the 5th day after surgery. Of the 11 patients, 5 have survived for more than 3 years (45.5%), and 4 have been disease-free for more than 3 years (36.3%).

**Conclusions:**

For liver tumours between the root angle of the middle and right hepatic veins, the modified median hepatic fissure approach is a safe and feasible method.

## Introduction

Surgical resection is the most effective and important method for the treatment of multiple types of malignant hepatic tumours [1]. The operation should take into account the resection margins and the safety of the operation. Ensuring sufficient negative incised margins at the tumour edge is a common method for radical resection, and, theoretically, it could reduce tumour recurrence. However, due to the particularity of hepatic surgery, blind pursuit of incised margins could mean the loss of non-tumour hepatic tissue and increase the risk of postoperative hepatic failure, especially when there is severe liver cirrhosis or the tumour is in a difficult location.

**Fig. 1:**
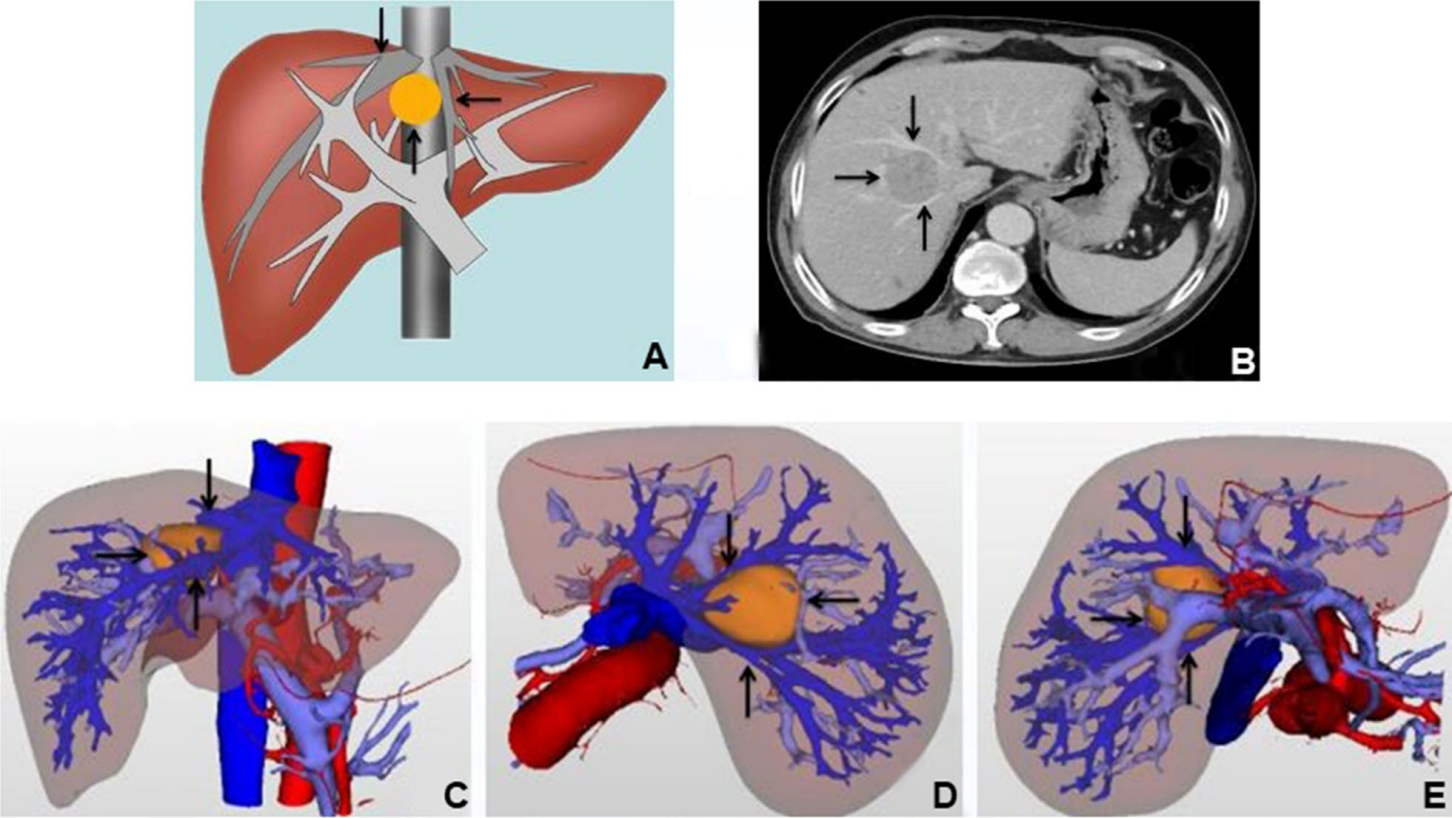
A schematic diagram of the structure of the tumor between the root angle of the hepatic middle and right veins and the typical CT images before operation.** A** Schematic diagram of tumor structure, the tumor (↑) as if embedded between the middle (←) and right (↓) hepatic veins; **B** abdominal CT showed tumor (→) the adjacent middle (↓) and right (↑) hepatic veins can be pushed and squeezed. Three-dimensional visualization reconstruction of liver before operation and the spatial structure relationship between tumor and middle and right hepatic vein: **C** positive view of three-dimensional reconstruction of liver: tumor (→), middle hepatic vein (↑), right hepatic vein (↓); **D** top view of three-dimensional reconstruction of liver: tumor (←), middle hepatic vein (↓), right hepatic vein (↑); **E** bottom view of three-dimensional reconstruction of liver: tumor (→), middle hepatic vein (↓), right hepatic vein (↑)

Liver tumours between the root angle of the middle and right hepatic veins are a special type of segment VIII tumour. These tumours are located in the deep part of segment VIII of the liver and are embedded between large blood vessels. Therefore, the tumour can push and squeeze the adjacent middle and right hepatic veins and even the inferior vena cava (Fig. 1A, B). In addition, because of the narrow anatomical space, there is little room for surgical operation. If performed improperly, resection can easily injure important surrounding blood vessels, leading to the risk of fatal massive bleeding or air embolism. According to the standard for radical resection of liver tumours, existing surgical resection approaches of liver segment VIII tumour are difficult to meet the requirement of sufficient residual liver volume while ensuring that negative resection margins for these tumours. Therefore, surgical resection of this type of tumour is still one of the difficulties in the field of hepatic surgery.

Transection of the median hepatic fissure has mainly been used in hemi-hepatectomy and hepatic caudate lobectomy. This approach can safely expose and separate important blood vessels such as the hepatic vein, portal vein, and inferior vena cava under direct vision, thereby allowing for safer removal of tumours. Here, we designed a modified median hepatic fissure approach for surgical resection of liver tumours located in the angle between the root of the middle and right hepatic veins and evaluated the safety and effectiveness of this surgical procedure.

### Methods

From April 2015 to November 2019, 11 patients with segment VIII tumours between the root angle of the middle and right hepatic veins underwent tumour resection using the modified median hepatic fissure approach in our centre. Patients were required to sign an informed consent form, and the procedure was approved by the Ethics Committee of the Second Affiliated Hospital of Nanchang University.

### Preoperative assessment

Indocyanine green (ICG) 15-min excretion and Child–Pugh classification were used to evaluate liver reserve function in all patients before surgery. Computed tomography (CT) and magnetic resonance imaging (MRI) were performed to assess the degree of liver cirrhosis and the spatial relationship between the tumour and important peripheral blood vessels. In order to accurately predict the surgical resection range and avoid injury to important vascular systems during surgery, we further used three-dimensional imaging techniques to display the complete shape and branch distribution of the intrahepatic vascular system and observe the stereoscopic relationship between the tumour and the intrahepatic vascular system based on different rotations and combination [2, 3] (Fig. 1C–E).

A preoperative multi-disciplinary team conference was held for all patients to determine the implementation of the operation. The criteria for surgical resection included good general condition, good liver function (Child–Pugh class A or B or ICG 15-min excretion rate less than 15%), and no intrahepatic main vascular cancer embolus or extrahepatic metastases.

### Operative procedure

The surgical procedure can be divided into the following four steps: (1) freeing the liver and blocking the hepatic blood flow; (2) intraoperative ultrasonography to determine the extent of the tumour and its relationship with peripheral blood vessels; (3) resection using the modified median hepatic fissure approach to isolate the parenchyma of the liver and expose and remove the tumour; and (4) treatment of the liver wound.

- Step 1: Right upper abdominal incision under the costal margin was performed, and suspended automatic retractors were used to fully expose the surgical field. The left and right perihepatic ligaments and all loose connective tissue in bare areas of the liver were cut, such that the liver was fully dissociated. Remove the left and right perihepatic ligaments and all loose connective tissues in the exposed area of the liver, so that the liver is completely free. Then, we first used the No. 10 ventricle drainage tube to bypass the hepatoduodenal ligament, prefabricated the blocking band in the first hepatic portal, and then freed the inferior inferior vena cava behind the first hepatic portal, and in the second liver under the diaphragm. Free the superior and inferior hepatic vena cava at the second hepatic hilum below the diaphragm, and preset blocking bands to block the blood flow into the liver or the whole liver if necessary to repair the ruptured hepatic vein or inferior vena cava.
- Step 2: Intraoperative ultrasonography was performed to further clarify the size, boundary, and depth of the tumour and its relationship with the surrounding adjacent vessels and check for microscopic tumours in other parts of the liver. The surface projections of the middle hepatic vein and the right hepatic vein were marked on the surface of the liver.
- Step 3: The hepatic capsule and superficial hepatic parenchyma were incised from the first porta hepatis to the second porta hepatis along the projection of the main route of the middle hepatic vein. The deep hepatic parenchyma was finely excised with CUSA, and the branches of the middle hepatic vein with a diameter of more than 1 mm were ligated and severed. When the surgical level was close to the lower edge of the tumour, the surgical path deviated from the median fissure of the liver and transected the liver parenchyma directly above the tumour, taking the upper pole of the tumour as the midpoint. Then, remaining close to the edge of the tumour, both sides of the tumour were carefully dissected to completely expose the root of the middle and right hepatic veins. Simultaneously, the surrounding tissue of the tumour was fully dissociated, and the tumour was removed (Figs. 2 and 3A–I). In this process, we routinely use controlled low central venous pressure technology during the operation to reduce the risk of hepatic vein bleeding. Combined with our pre-indwelling hepatic portal block tape, once hepatic vein injury and bleeding occurs, We will repair the hepatic vein breach when the blood flow to the liver is blocked or the blood flow to the whole liver is blocked.
  **Fig. 2:**
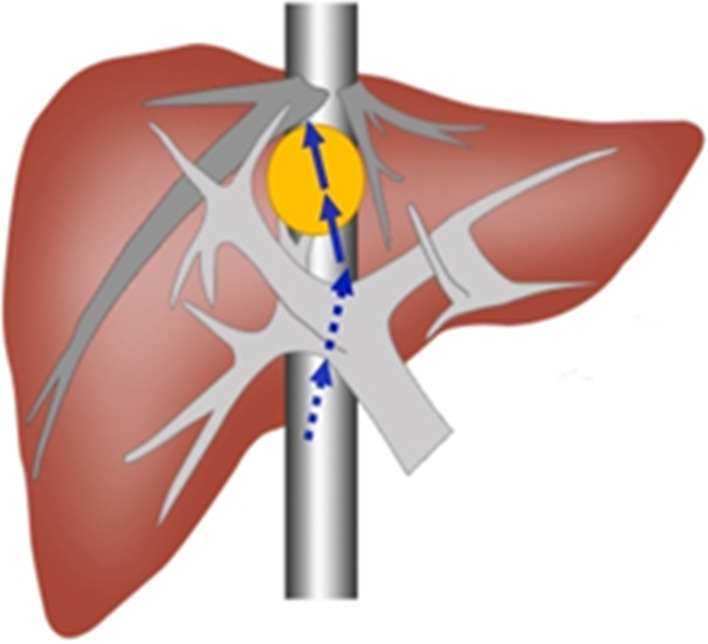
Surgical schematic diagram. Schematic diagram of liver parenchyma split by modified median hepatic fissure and combined with tumor resection
  **Fig. 3:**
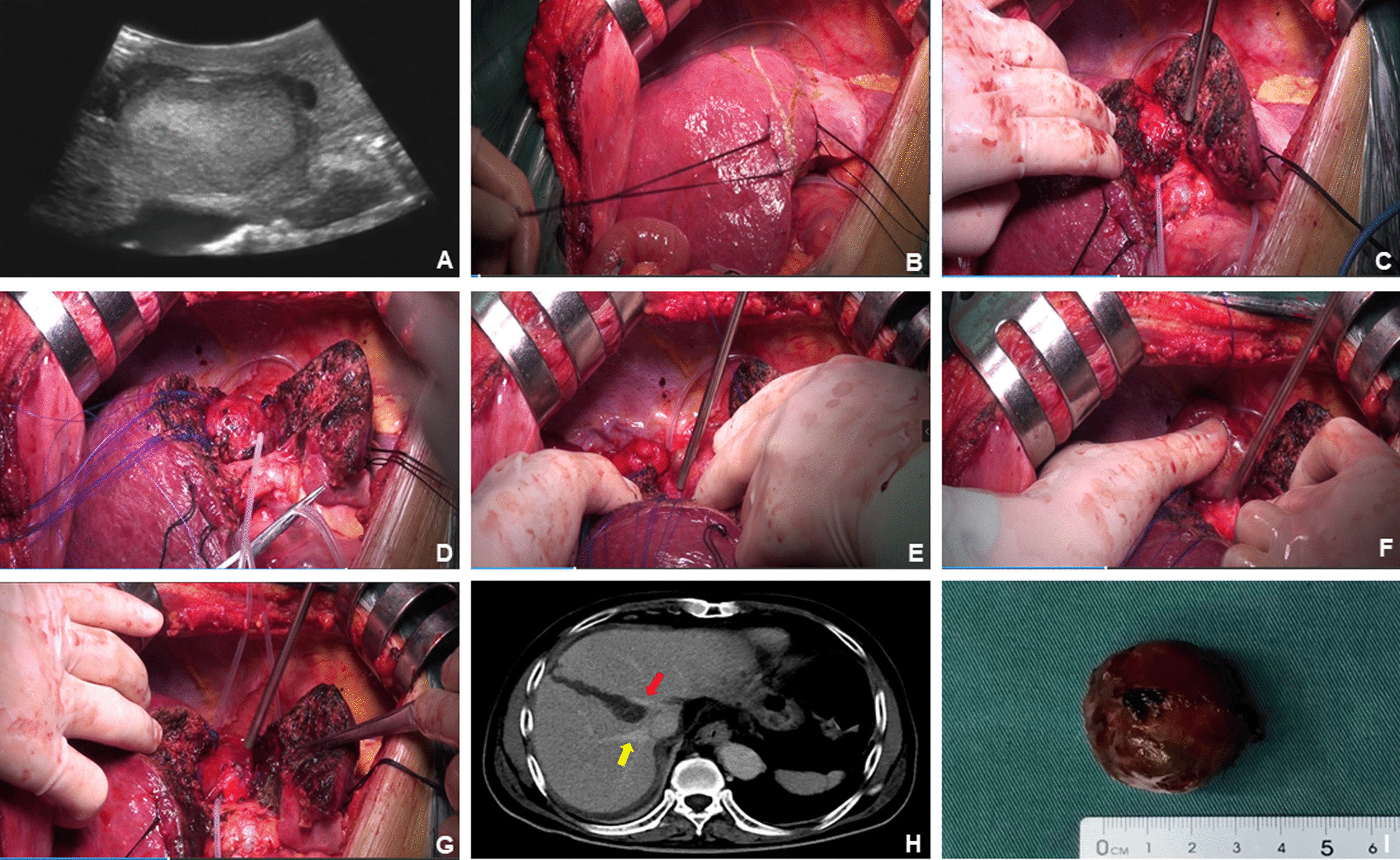
Intraoperative and postoperative imaging of liver parenchyma split by modified median hepatic fissure and combined with tumor resection.** A** Intraoperative ultrasound suggested that the tumor was between the root angle of the middle and right hepatic veins; **B** intraoperative surface projections of tumor, middle hepatic vein and right hepatic vein marked on the surface of the liver. **C** When the operation is close to the lower margin of the tumor, split the liver parenchyma from the front of the tumor; **D** tumor and middle hepatic vein; **E** tumor and right hepatic vein; **F** tumor and postcaval vein; **G** surgical field after resection of tumor; **H** CT imaging on the 7th day after operation, yellow arrow: right hepatic vein, red arrow: middle hepatic vein; **I** resection specimens of liver cancer
- Step 4: The liver wounds were carefully examined for bile leakage and active bleeding. Then the wounds were sprayed with fibrin glue and sutured to reduce postoperative wound bleeding and prevent the formation of hematoma. Hepatic inflow and outflow were confirmed.

### Postoperative complications and follow-up

Postoperative hepatic insufficiency was defined using the ‘50–50 criteria’ of serum total bilirubin (TB) and prothrombin time (PT) on the 5th postoperative day [4]. According to the definition of the International Study Group of Liver Surgery, postoperative bile leakage was defined as bilirubin in drainage-fluid three times higher than the upper limit of normal TB. According to severity grading of bile leakage after hepatobiliary and pancreatic surgery by the ISGLS, postoperative bilirubin leakage was divided into Grade A, B, and C [5]. The Clavien–Dindo score was used for the grading of all complications, and we recorded the highest level of complication per patient [6].

All the patients received liver function tests, tumour marker examinations, abdominal ultrasonography, and thoracic-abdominal CT every 3 months in the first year after surgery and semi-annually thereafter. Re-examination of HBV-DNA was required for patients with hepatitis B viral infection. Disease-free survival (DFS) and overall survival (OS) were defined as the time between the date of hepatectomy and the date of diagnosis of tumour recurrence and the date of death, respectively. The final follow-up date for this study was October 2020. This study was conducted and reported in line with the STROCSS criteria (www.strocssguideline.com) [7].

## Results

In our centre, 11 patients with this type of tumour underwent this new method of surgery. Of these 11 patients, 8 were men and 3 were women. The patient age ranged from 56 to 74 years (average age, 63.5 years). Nine patients had hepatocellular carcinoma, and 2 patients had colorectal liver metastases (CRLM). Six of 9 patients with primary hepatocellular carcinoma were positive for HBV-DNA and received regular anti-viral therapy before surgery.

The average operation time was 196 min (160–240 min), and the average intraoperative blood loss was 318 mL (260–450 mL). One patient with CRLM lost 450 mL of blood, which may be related to the corresponding vascular inflammatory reaction caused by long-term chemotherapy before surgery. Intraoperative blood loss of 400 mL also occurred in one patient with primary liver cancer. This patient had obvious liver cirrhosis, and the patient had errhysis during surgery. Further, the tumour was close to the inferior vena cava, and it was difficult to separate, so the hepatic blood flow was blocked. Other patients had less intraoperative bleeding, and 4 patients received intraoperative blood products.

**Fig. 4:**
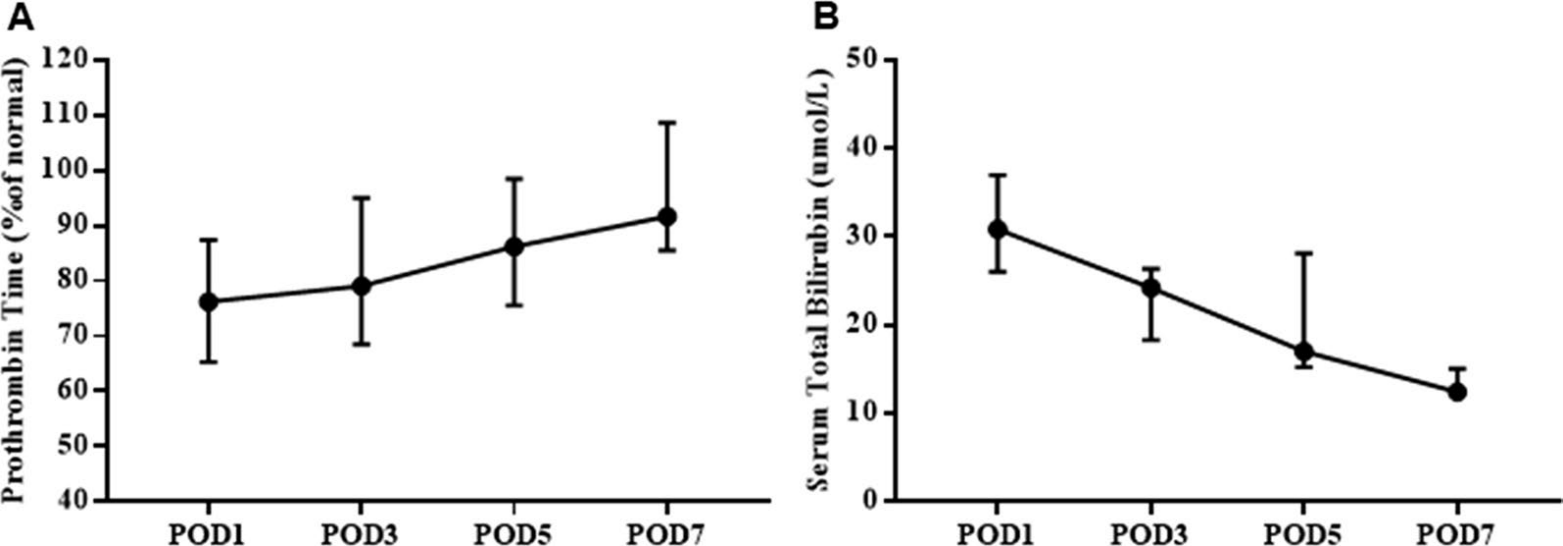
Postoperative liver function level of patients.** A** Postoperative prothrombin time (PT) level; **B** postoperative total serum bilirubin level (TB) level

The postoperative TB and PT in all patients are shown in Fig. 4. On the 1st postoperative day, the median PT was 76.2% (range, 51–97%), and the median TB was 30.84 µmol/L (range, 21.91–47.66 µmol/L). The PT showed a gradual upward trend over time, and the TB showed a gradual downward trend. The liver function and coagulation function of most patients returned to the normal range by the 7th day after surgery.

Two patients developed grade A bile leakage after surgery and recovered after continuous unobstructed drainage. Two patients developed secondary pleural effusion after surgery and were cured after symptomatic treatment. No postoperative complications exceeding Clavien–Dindo grade II, such as intra-abdominal haemorrhage and liver failure, occurred in any of the patients.

All 11 patients had long-term follow-up, the median follow-up interval was 37 months (range, 13–66 months). Two patients with CRLM developed lung metastasis at 26 months and 35 months after hepatectomy (18%), 1 patient with HCC developed bone metastasis at 20months after hepatectomy (9%), and 2 patients with HCC developed intrahepatic metastasis at 46 months and 50 months after hepatectomy (18%). All patients with recurrence after surgery are currently receiving anti-tumour therapy. At present, 5 patients have survived for more than 3 years (45.5%), and 4 have survived disease-free for more than 3 years (36.3%) (Table 1).

**Table 1:** Perioperative period results and follow-up data of all 11 patients

No.	Sex/age	Operation date	Disease	Tumor size (mm)	Operation time (min)	Vascular clamp	Blood loss (mL)	RBC transfusion (units)	Postoperative complications	Hospital stay (d)	DFS	OS
1	M/58	2015.04	HCC	45	230	PTC for 20 min	360	1	None	13	3 years 10 months Intrahepatic recurrence	5 years 2 months
2	M/63	2015.05	HCC	40	210	TVE for 15 min	400	1.5	Pleural effusion	12	No recurrence 5 years 5 months	–
3	M/56	2016.03	HCC	35	180		280		None	10	4 years 2 months Intrahepatic recurrence	–
4	M/74	2016.10	CRLM	25	240	PTC for 15 min + 12 min	450	2	Grade A bile leaks	14	2 years 11 months Lung metastases	3 years 9 months
5	M/59	2017.05	HCC	33	170		230		Wound hematoma	16	1 years 8 months Bone metastases	2 years 4 months
6	M/73	2017.09	HCC	47	160		260		None	7	No recurrence 3 years 1 months	–
7	W/71	2018.02	HCC	40	180		340	1	None	9	No recurrence 2 years 8 months	–
8	W/58	2018.04	CRLM	28	200	PTC for 17 min	280		None	8	2 years 2 months Lung metastases	–
9	M/67	2018.09	HCC	38	160	PTC for 10 min	220		Pleural effusion	10	No recurrence 2 years 1 months	–
10	M/56	2019.02	HCC	30	180	PTC for 12 min	200		Grade A bile leaks	13	No recurrence 1 years 8months	–
11	W/63	2019.9	HCC	34	160	PTC for 15 min	150		None	8	No recurrence 1 years 1 months	–

*PTC* portal triad clamping. *TVE* total vascular exclusion

## Discussion

Segment VIII of the liver is located at the top of the diaphragm above the anterior lobe of the right liver. The right hepatic vein is to the right of this segment, the middle hepatic vein is to the left, and the inferior vena cava is found deep within the segment. In addition, the portal vein and the right branches of the hepatic artery also extend and connect in this segment of the liver. Therefore, segment VIII is the segment most adjacent and closely related to the great vessels.

Liver tumours between the root angle of the middle and the right liver veins are a special type of segment VIII tumours, and choosing a reasonable surgical resection scheme is the key to treatment. According to the standard for radical resection of liver tumours, the incisal margin should be more than 1 cm from the tumour [8]. However, right anterior hepatectomy (segments V and VIII) and right hemi-hepatectomy cannot guarantee negative margins around the middle hepatic vein, and middle hepatectomy (segments IV, V, and VIII) and enlarged left hepatectomy (segments II, III, IV, V, and VIII) cannot guarantee negative margins around the right hepatic vein [9]. Extended right hepatectomy can achieve R0 resection; however, this approach also has some limitations. About 80% of patients with primary liver cancer and some patients with metastatic liver tumours have liver cirrhosis, chronic hepatopathy, or liver tissue injury caused by comprehensive tumour treatment. In these patients, poor hepatic functional reserve often leads to insufficient residual hepatic function after resection because too much normal liver parenchyma was resected, which increases the risk of postoperative liver failure and other serious complications [10, 11]. Retaining more normal hepatic tissue than what is preserved during extensive hepatectomy is vital among patients with hypohepatia. Although extended hepatectomy could be tolerated in patients without liver cirrhosis and good hepatic functional reserve, it leads to unnecessary loss of normal hepatic tissues and is not conducive to treatment after tumour recurrence. Further, large-scale hepatectomy leaves a large wound that takes time to heal, and it is easy to damage the adjacent important blood vessels and bile duct branches during surgery, which increases the risk of hepatic ischemia, necrosis, and bile leakage [12]. In addition, if non-surgical methods such as radiofrequency ablation or microwave therapy are administered, they can damage the middle and right hepatic veins by the heat conduct effect and incomplete ablation caused by the heat sink effect, which can decrease their antitumor effect, thus, it is necessary to seek a suitable surgical approach for liver tumours located in the angle between the root of the middle and right hepatic veins.

Transection of the median hepatic fissure has been mainly used in hemi-hepatectomy and hepatic caudate lobectomy. Yamamoto et al. reported the first hepatic caudate lobectomy by dissecting the median fissure of the liver in 1992 [13]. For tumours between the root angle of the middle and right hepatic veins, a modified median hepatic fissure approach is used to remove the tumour. This surgical method expands the space to expose the surgical visual field and converts a deep tumour resection to a more superficial tumour resection. This technique has several advantages. First, the important blood vessels, such as the hepatic vein, portal vein, and inferior vena cava, can be safely exposed and separated under direct vision, and there is better exposure for surgery. Second, when the liver is cut along the median fissure, there is no important plumbing system in that section of the hepatic parenchyma. Third, the tumour is rarely squeezed during hepatectomy, reducing the risk of iatrogenic tumour shedding or transvascular metastasis. Last, this path increases the exposure on both sides of the tumour, which shortens the unilateral resection distance and is more conducive to accurate resection under the middle and right hepatic veins under direct vision. This method is particularly beneficial to repair the vascular rupture under direct vision in the event of injury.

Although some studies have suggested that sufficient surgical margins may be helpful in preventing postoperative recurrence of liver cancer [14–16], many studies have shown that there is no correlation between the extent of resection and postoperative recurrence or long-term prognosis. Poon et al. found that portal vein diffusion and multicentric liver cancer are the main causes of postoperative recurrence of liver cancer. If extensive hepatectomy does not effectively reduce the risk of postoperative recurrence of liver cancer, then tumours that do not have a 1-cm surgical margin should not be contraindicated for hepatectomy [17, 18]. Matsui et al. in an analysis of nearly 470 cases of liver cancer, proved that when the tumour was adjacent to the main blood vessels of the liver, there was no significant difference in DFS and OS between patients who underwent R0 resection and those who underwent R1/R2 resection in the same period [19]. Some scholars have suggested that when the patient’s liver reserve function is limited, the preservation of sufficient liver parenchyma is more important than a sufficient surgical margin. Of the 11 patients in this study, 5 (45.5%) survived for more than 3 years, and 4 were disease-free for more than 3 years (36.3%). These results are similar to the prognostic data reported in recent years for isolated resection of liver segment VIII tumours [9, 20, 21].

## Conclusions

In summary, liver tumours between the root angle of the middle and right hepatic veins are a special type of segment VIII tumour, and the surgical resection method must be different from that for conventional segment VIII tumours. The modified median hepatic fissure approach described in this study can completely resect the tumour while retaining the maximum normal hepatic parenchyma. Therefore, the physiological function of the liver can be preserved to the maximum extent, this modified median hepatic fissure approach is especially suitable for patients with benign liver tumors between the middle and right vein roots, HCC with intact capsule and no satellites, isolated colonic liver metastases, or liver malignant tumors whose residual liver volume is insufficient and cannot tolerate large-scale hepatectomy. However, it is necessary to use this technique with caution for non-enveloped liver malignancies that are between the middle and right vein roots of the liver. At the same time, there were several inevitable limitations in our study. To begin with, this study was limited by its retrospective nature, and selection bias could not be completely avoided. In addition, the sample size of this study is relatively small, so multicenter study on large sample size are needed to validate the advantages of modified median hepatic fissure approach for resection of liver tumours located in the angle between the root of the middle and right hepatic veins in the future.

## Data Availability

Not applicable.

## Abbreviations

ICG: Indocyanine green
CT: Computed tomography
MRI: Magnetic resonance imaging
TB: Total bilirubin
PT: Prothrombin time
DFS: Disease-free survival
OS: Overall survival
CRLM: Colorectal liver metastases

## References

[1] Yang JD, Hainaut P, Gores GJ, Amadou A, Plymoth A, Roberts LR (2019). A global view of hepatocellular carcinoma: trends, risk, prevention and management. Nat Rev Gastroenterol Hepatol.

[2] Tian F, Wu J-X, Rong W-Q, Wang L-M, Wu F, Yu W-B (2015). Three-dimensional morphometric analysis for hepatectomy of centrally located hepatocellular carcinoma: a pilot study. World J Gastroenterol.

[3] Mise Y, Tani K, Aoki T, Sakamoto Y, Hasegawa K, Sugawara Y (2013). Virtual liver resection: computer-assisted operation planning using a three-dimensional liver representation. J Hepatobliary Pancreat Sci.

[4] Balzan S, Belghiti J, Farges O, Ogata S, Sauvanet A, Delefosse D (2005). The “50–50 criteria” on postoperative day 5: an accurate predictor of liver failure and death after hepatectomy. Ann Surg.

[5] Birgin E, Tesfazgi W, Knoth M, Wilhelm TJ, Post S, Rückert F (2018). Evaluation of the new ISGLS definitions of typical posthepatectomy complications. Scand J Surg.

[6] Bolliger M, Kroehnert JA, Molineus F, Kandioler D, Schindl M, Riss P (2018). Experiences with the standardized classification of surgical complications (Clavien–Dindo) in general surgery patients. Eur Surg.

[7] Agha R, Abdall-Razak A, Crossley E, Dowlut N, Iosifidis C, Mathew G (2019). STROCSS 2019 guideline: strengthening the reporting of cohort studies in surgery. Int J Surg.

[8] Zhou XP, Quan ZW, Cong WM, Yang N, Zhang HB, Zhang SH (2007). Micrometastasis in surrounding liver and the minimal length of resection margin of primary liver cancer. World J Gastroenterol.

[9] Kishi Y, Hasegawa K, Kaneko J, Aoki T, Beck Y, Sugawara Y (2012). Resection of segment VIII for hepatocellular carcinoma. Br J Surg.

[10] Kanda T, Goto T, Hirotsu Y, Moriyama M, Omata M (2019). Molecular mechanisms driving progression of liver cirrhosis towards hepatocellular carcinoma in chronic hepatitis B and C infections: a review. Int J Mol Sci.

[11] Duwe G, Knitter S, Pesthy S, Beierle AS, Bahra M, Schmelzle M (2017). Hepatotoxicity following systemic therapy for colorectal liver metastases and the impact of chemotherapy-associated liver injury on outcomes after curative liver resection. Eur J Surg Oncol.

[12] Kokudo T, Hasegawa K, Yamamoto S, Shindoh J, Takemura N, Aoki T (2014). Surgical treatment of hepatocellular carcinoma associated with hepatic vein tumor thrombosis. J Hepatol.

[13] Yamamoto JKT, Shimada K, Yamasaki S, Takayama T, Makuuchi M (1999). Anterior transhepatic approach for isolated resection of the caudate lobe of the liver. World J Surg.

[14] Lee WC JL, Chen MF (2002). Estimation of prognosis after hepatectomy for hepatocellular carcinoma. Br J Surg.

[15] Shi M, Guo R-P, Lin X-J, Zhang Y-Q, Chen M-S, Zhang C-Q (2007). Partial hepatectomy with wide versus narrow resection margin for solitary hepatocellular carcinoma. Ann Surg.

[16] Shimada K, Sakamoto Y, Esaki M, Kosuge T (2008). Role of the width of the surgical margin in a hepatectomy for small hepatocellular carcinomas eligible for percutaneous local ablative therapy. Am J Surg.

[17] Poon RT, Fan ST, Ng IO (2000). Significance of resection margin in hepatectomy for hepatocellular carcinoma: a critical reappraisal. Ann surg.

[18] Nara S, Shimada K, Sakamoto Y, Esaki M, Kishi Y, Kosuge T (2012). Prognostic impact of marginal resection for patients with solitary hepatocellular carcinoma: evidence from 570 hepatectomies. Surgery.

[19] Matsui Y, Terakawa N, Satoi S, Kaibori M, Kitade H, Takai S (2007). Postoperative outcomes in patients with hepatocellular carcinomas resected with exposure of the tumor surface: clinical role of the no-margin resection. Arch Surg.

[20] Yu YQ, Tang ZY, Ma ZC, Zhou XD, Mack P (1993). Resection of segment VIII of liver for treatment of primary liver cancer. Arch Surg.

[21] Yu YQ, Zhou XD, Tang ZY, Xu DB, Feng XS (1993). Experience with resection of segment VIII of liver for hepatocellular carcinoma. Semin Surg Oncol.

